# First Demonstration of Cardiac Resynchronization Therapy Defibrillator Service Life Exceeding Patient Survival in a Heart Failure with Reduced Ejection Fraction Cohort

**DOI:** 10.19102/icrm.2020.111203

**Published:** 2020-12-15

**Authors:** Jeffrey L. Williams, Bertha Harley, Gabriella Williams

**Affiliations:** ^1^Lakeland Regional Health System, Lakeland, FL, USA; ^2^University of Notre Dame, Notre Dame, IN, USA

**Keywords:** Battery, cardiac synchronization therapy, implantable cardioverter defibrillator, longevity, replacement

## Abstract

The occurrence of patient longevity exceeding implantable cardioverter-defibrillator (ICD) service life has important implications for patient outcomes and the cost of care. Battery capacity as measured in ampere-hours (Ah) is a strong predictor of survival to an elective replacement indicator (ERI) point and 2.1 Ah is the largest-capacity ICD battery in use at our facility. This was a long-term study of ICDs out of service (OOS) in patients with heart failure with reduced ejection fraction who received a 2.1-Ah cardiac resynchronization therapy defibrillator (CRT-D). All 2.1-Ah CRT-D systems implanted (n = 418) from August 1, 2008 through August 31, 2016 were included in this retrospective chart review. The primary endpoint was device OOS due to the battery reaching an ERI point, patient death, infection/erosion, advisory/recall, heart transplant, or unspecified. The maximum follow-up period was 10.3 years, with a mean follow-up length of 4.7 years. The most common reason for device OOS was patient death (65.6%), with only 5.7% of devices reaching the ERI point during the study. There was a period of OOS acceleration driven numerically by patient death in the sixth to ninth years of follow-up. Male sex, ischemic cardiomyopathy, elevated creatinine level, advanced age, and reduced ejection fraction were associated with OOS (p < 0.05). To our knowledge, this is the first study to report ICD battery life exceeding patient survival in a chronic heart failure cohort. During an accelerated time of CRT-D OOS (when it is expected that ~98% of 1.0-Ah and 1.4-Ah CRT-D systems reach an ERI point), patient death resulted in substantially more device OOS than battery replacement and avoided costs of complications and generator changes. These results help to explain the elevated risks of CRT-D generator changes in shorter-longevity devices.

## Introduction

The timing and reason for cardiac resynchronization (CRT) defibrillator (CRT-D) devices being out of service (OOS) has important implications for patient outcomes and the cost of care. This study examines patient and device characteristics that result in device OOS. There has been a mismatch between the service life of implantable cardioverter-defibrillators (ICDs) and patient longevity that has been suggested to pose a significant clinical and economic burden that must be addressed.^1^ Indeed, the largest study to date examining this interplay between patient survival and ICD battery longevity^2^ suggests that, despite the introduction of improved battery technology, the survival of patients is better than ICD longevity and not vice versa as should be the case. Von Gunten et al. concluded in 2016 that overall ICD longevity continued to be 70% at five years, whereas patient survival was 80% at the same time point.^2^ These authors additionally suggested that a marked improvement in battery technology with a transition to a 2.1–ampere-hour (Ah) lithium–manganese battery may reverse this mismatch but cautioned that more data were needed. These points were recently echoed by Boriani et al.,^3^ who concurred that patient survival still exceeds device longevity while suggesting that extending device longevity further could reduce complications, comply with patient preferences, and improve the cost-effectiveness. Device longevity has been shown to have the largest impact on the cost-effectiveness of ICD therapy by a reduction in device replacements, hospitalizations, and complications.^3,^^4^

Clearly, one can see that an average battery life of less than the typical survival length of an ICD patient might expose them to a procedure during a very vulnerable period in their life (ie, a generator change performed very near to the patient’s physiologic end of life). Indeed, we and others have demonstrated that there are substantial differences in device longevity.^5–8^ Furthermore, it is clear that generator replacements offer a substantial risk of major complications (4%–9%).^9,10^ The present study is a long-term evaluation of OOS in a contemporary cohort of patients with heart failure with reduced ejection fraction (EF) (HFrEF) who received 2.1-Ah CRT-D systems. Our hypothesis is that the improved battery longevity demonstrated in a “real-world” outcomes analysis of exclusively 2.1-Ah battery technology would reverse the longstanding trend of patient survival exceeding CRT-D battery longevity.

## Methods

### Patient selection

This retrospective chart review was approved by the institutional review board of Lakeland Regional Health System and the study received a waiver of the need to collect informed consent. All 2.1-Ah CRT-D systems implanted at our center from August 1, 2008 through August 31, 2016 were included in this retrospective chart review. For each patient, the decision to implant an ICD was made by the physician caring for the patient at that time according to standard clinical care indications. The use of a remote CRT-D monitoring system (Latitude; Boston Scientific, Natick, MA, USA) was used to identify a cohort of patients for the chart review and baseline demographics and device data were obtained from the electronic medical records. Final outcomes were evaluated through February 21, 2019. The primary endpoint was device OOS due to the battery reaching an elective replacement indicator (ERI) point, patient death, infection/erosion, advisory/recall, heart transplant, or unspecified (including device deactivation).

### Data analysis

The offline analysis consisted of a retrospective chart review to obtain baseline demographics, device data, and survival obtained from the electronic medical records and a remote monitoring system in a similar fashion to a prior study^5^ of the authors that examined ICD battery longevity. The data elements were evaluated using Matlab (The Mathworks Inc., Natick, MA, USA) and the Statistical Package for the Social Sciences (IBM Corp., Armonk, NY, USA). The final device OOS timing was obtained. Categorical variables are presented as n (%). Continuous variables are expressed as mean ± standard deviation (SD). Baseline clinical characteristics were compared between patients with devices that reached OOS and those with active devices during follow-up using the chi-squared test for dichotomous variables and one-way multivariate analysis of variance or Tukey post-hoc test as appropriate. Kaplan–Meier curves were plotted for the time to CRT-D OOS and compared using the log-rank test. Kaplan–Meier estimation of the survival function for CRT-D systems reaching OOS from the observed survival times was performed without the assumption of an underlying probability distribution.^11^

## Results

A total of 418 patients were included for analysis; complete demographic data were available for 99% of this group. The maximum possible follow-up was 10.3 years and the mean follow-up was 4.7 years ± 2.7 years. The average age at implantation was 72.3 years ± 10.8 years. The average creatinine level was 1.3 mg/dL ± 0.6 mg/dL. The average EF was 27% ± 10%. One hundred ninety-one patients had nonischemic cardiomyopathy (45.7%), 223 patients were considered to have ischemic cardiomyopathy (53.3%), and four patients (1%) had undefined cardiomyopathy. One hundred and forty-six patients were female (34.8%) and 272 patients were male (65.2%). During the mean follow-up period of 4.7 years ± 2.7 years, there were 105 patient deaths.

**Figure 1:**
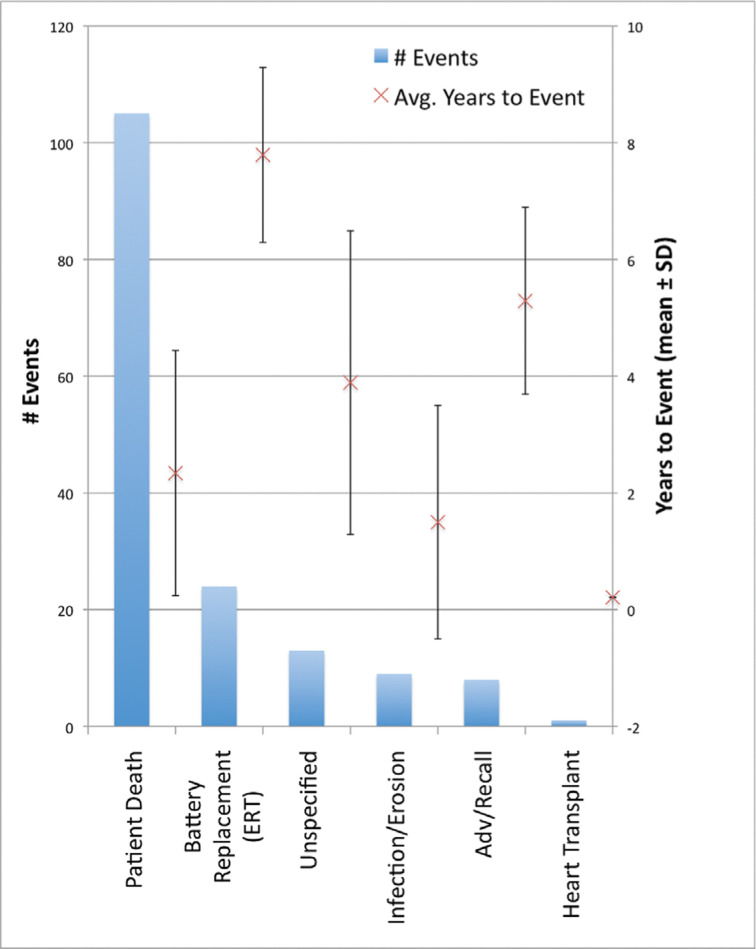
Mode and Timing to OOS. The primary endpoint for device OOS occurred in 160 patients during the study. Among these OOS patients (shown in bars), death occurred in 105 patients (65.6%), the battery reached ERI in 24 patients (15%), infection/erosion occurred in nine patients (5.6%), advisory/recall occurred in eight patients (5%), heart transplant occurred in one patient (0.6%), and an unspecified event occurred in 13 patients (8.1%). The years to event (mean ± SD) is shown with SD bars. OOS: out of service; ERI: elective replacement indicator; SD: standard deviation.

The primary endpoint for device OOS occurred in 160 patients during the study. Of these OOS patients, the battery reached the ERI point in 24 patients (15%), patient death occurred in 105 patients (65.6%), infection/erosion occurred in nine patients (5.6%), advisory/recall occurred in eight patients (5%), heart transplant occurred in one patient (0.6%), and the cause was unspecified in 13 patients (8.1%). The mode and average time to occurrence of OOS are shown in **Figure 1** and **Table 1**. The most common reason for device OOS was patient death (105/160 patients; 65.6%); the average time to patient death was 2.3 years ± 2.1 years. During the entire study, 5.7% of devices reached the ERI point (average time to ERI: 7.8 ± 1.5 years). Overall, a patient survival rate of 74.9% during the mean follow-up period of 4.7 years ± 2.7 years was observed. There were no devices that achieved OOS due to ERI at a point sooner than the mean follow-up time of 4.7 years.

**Figure 2:**
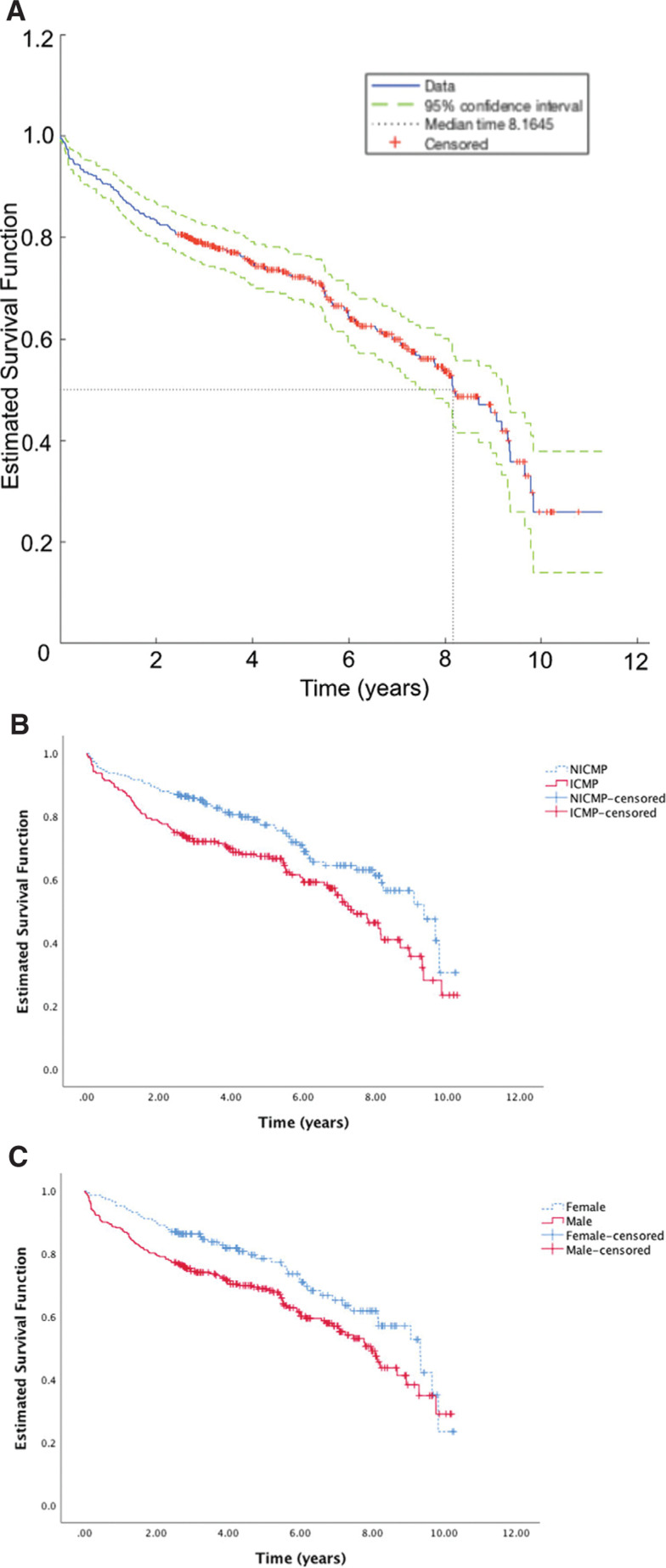
**A:** Kaplan–Meier estimation of the survival function for CRT-D systems reaching OOS. The primary endpoint for device OOS occurred in 160 patients during the study. The median time for 50% survival was 8.16 years. There was an acceleration in OOS during the sixth through ninth years of follow-up. **B:** Kaplan–Meier estimation of the survival function for CRT-D systems reaching OOS stratified by type of cardiomyopathy. The cumulative survival function was significantly lower in patients with ischemic cardiomyopathy (p = 0.005, log-rank test), which carried through the follow-up period. **C:** Kaplan–Meier estimation of the survival function for CRT-D systems reaching OOS stratified by sex. The cumulative survival function was significantly lower in male patients (p = 0.025, log-rank test), although the curves crossed over at end of follow-up. ICMP: ischemic cardiomyopathy; NICMP: nonischemic cardiomyopathy.

Kaplan–Meier survival curves for the time to CRT-D OOS are shown in **Figure 2**. **Figure 2A** presents the overall survival function including 95% confidence intervals (CIs) with a median time to OOS of 8.2 years. Notably, there was a period of OOS acceleration driven by patient death from the sixth to ninth years of follow-up. Meanwhile, the Kaplan–Meier survival curve for the time to CRT-D OOS in patients with ischemic versus nonischemic cardiomyopathy is shown in **Figure 2B**. The cumulative time to OOS survival function was significantly lower among patients with ischemic cardiomyopathy (p = 0.005). Finally, the Kaplan–Meier survival curve for the time to CRT-D OOS in male versus female patients is shown in **Figure 2C**. The cumulative survival function was significantly lower in male patients (p = 0.025). Kaplan–Meier curves were compared using the log-rank test **(Table 2)**. Chi-squared tests for dichotomous variables showed a statistically significantly lower cumulative survival time to OOS for male patients [hazard ratio (HR): 0.918; 95% CI: 6.08–7.06; p = 0.025] and those with ischemic cardiomyopathy (HR: 0.728; 95% CI: 5.85–6.9; p = 0.005). One-way multivariate analysis of variance of devices reaching OOS versus active devices showed a statistically significant effect of advanced age (HR: 1.03; 95% CI: 72.8–76.1; p = 0.005), elevated creatinine level (HR: 1.55; 95% CI: 1.3–1.5; p = 0.031), and lower EF (HR: 0.045; 95% CI: 0.2–0.27; p = 0.008).

## Discussion

Patient deaths (mean time to death: 2.3 ± 2.1 years) was a more frequent cause of CRT-D OOS than ERI (mean time to ERI: 7.8 ± 1.5 years). This study revealed in our HFrEF cohort that the use of a 2.1-Ah battery resulted in a reversal of the longstanding mismatch between device longevity and patient survival. We observed an acceleration of CRT-D OOS frequency during the sixth through ninth years of follow-up (when it is expected that ~98% of 1.0-Ah and 1.4-Ah CRT-D systems reach ERI); during this time, patient deaths (n = 105; 65.6%) resulted in numerically more instances of device OOS than battery replacements did (n = 24, 15%). Device longevity exceeding patient survival addresses a known clinical and economic burden^1^ and may result in fewer complications, increase the cost-effectiveness, and would be more in line with patient preferences.^3^

Prior to 2008, the 2.1-Ah CRT-D battery was only available in silver vanadium oxide (SVO) with the extended longevity option. The SVO battery had increased charge times due to impedance issues, causing the ERI to trip.^12^ In 2008, this battery chemistry was changed to lithium–manganese dioxide (Li-MnO_2_), with the 2.1-Ah model becoming a standard option. CRT-D battery capacity as measured in Ah was shown to be a strong predictor of survival to ERI^6^ and the 2.1-Ah Li-MnO_2_ battery is the largest-capacity ICD battery in use at our facility. The total battery capacity in our study is reported as 2.1 Ah based upon Munawar et al.’s call to standardize the reporting of defibrillator battery longevities.^4^

There are limited data on the survival of patients with CRT-D; however, CRT has been shown to offer improved outcomes in terms of both functional capacity and EF. Five-year patient survival rates have been estimated at 60% to 89% based upon underlying patient characteristics.^13–15^ However, population-based estimates of CHF patient survival have shown lower survival rates. In the United Kingdom, the five-year survival rate was found to range from 40% to 60% depending on whether or not the patient was hospitalized with CHF.^16^ To our knowledge, however, this is the first study showing CRT-D battery longevity exceeding patient survival despite our 74.9% survival rate of patients with an average EF of 27% ± 10% during the mean follow-up period of 4.7 ± 2.7 years.

The risk of ICD generator-change complications is not trivial and it is reasonable that clinicians should attempt to minimize the need to undergo device replacement.^17^ The Medicare rate of device infection is 2%.^18^ Extraction (necessitated by an infection) has been associated with a 1% to 2% major complication rate as well as a cost per episode of $24,459 ± $14,585.^9^ Furthermore, there is a 1% rate of hematoma requiring evacuation with a cost per episode of $6,187 ± $2,631. Finally, generator change is associated with a five-fold higher risk of lead alert and/or lead failure.^19^ All in all, it is reasonable to estimate a major complication rate as high as 4% to 9% is associated with ICD generator changes.^9,10^ Extended battery longevity that exceeds patient survival as we have demonstrated may decrease costs and complications by avoiding the need for generator changes in HFrEF patients. Certainly, device longevity issues should be a factor in device selection from both the perspectives of the provider and patient.^3^ A majority of patients have expressed a preference for longer battery longevity rather than a smaller device size to reduce the number of replacement procedures.^20^

### Natural history of contemporary cardiac resynchronization therapy defibrillator patients

Our study offers more insight into the natural history of contemporary 2.1-Ah CRT-D systems with respect to HFrEF patient outcomes. We found an 8.2-year median survival to OOS with an acceleration in OOS occurring between the sixth and ninth years of the study period. Female and nonischemic cardiomyopathy patients had higher survival rates. The mean follow-up of 4.7 years combined with only 5.7% of devices (24/418 patients) reaching ERI (average time to ERI: 7.8 years) during the study is consistent with findings of other reports suggesting the improved longevity of recent-generation 2.1-Ah CRT-D batteries.^5–8,21^ Indeed, it is expected that approximately 98% of 1.0-Ah and 1.4-Ah CRT-D systems will reach ERI between six and nine years after implantation.^22^ Our 4.7-year patient survival and overall 2.1-Ah ICD longevity rates were 75% and 100%, while, in 2016, the average five-year patient survival and overall ICD longevity rates were 80% and 70%, respectively.^2^

Advanced age (HR: 1.03; 95% CI: 72.8–76.1; p = 0.005), elevated creatinine (HR: 1.55; 95% CI: 1.3–1.5; p = 0.031), and lower EF (HR: 0.045; 95% CI: 0.24–0.27; p = 0.008) were associated with CRT-D OOS, which was numerically driven by patient death. Device replacement not only increases the risk of complications but also the total cost of therapy given the additional procedures required and extra costs associated with managing replacement-related complications.^21^ The longevity of CRT-D batteries has been found to be shorter than that of single- and dual-chamber defibrillator batteries^23^ and, historically, ERI has been the most frequent cause of device replacement reported in prior studies.^2,24,25^ To our knowledge, our study is the first to report patient deaths as a more frequent cause of CRT-D OOS (mean time to death: 2.3 ± 2.1 years) rather than ERI (mean time to ERI: 7.8 ± 1.5 years), likely due to our focus on 2.1-Ah battery CRT-D systems.

### Socioeconomic impact of battery longevity

CRT-D replacement due to battery depletion is a significant cost-driver for payors^21,26^ and a significant complication-driver for patients.^9,18^ Landolina et al. found the need for device replacements at six years was reduced from 83% to 68% with the use of devices with improved battery longevity from the most recent generation.^21^ This value (from both cost and quality standpoints) of increased device longevity has led the National Institute for Health and Care Excellence (NICE), which offers guidance to the National Health Service (NHS) of the United Kingdom, to suggest that the 2.1-Ah devices with extended battery life may offer “clinical and patient benefit[s] and [be] associated with fewer replacement procedures… [they] may save between £2,120 and £5,627 per patient over 15 years through a reduction in the need for replacement procedures. This could save the NHS in England around £6 million in the first five years.”^27^ A conservative estimate of the current annual volume of CRT-D implants in the United States can be estimated at 27,605 based upon a serial cross-sectional study using the National Inpatient Sample database between 2006 and 2012.^28^ Using similar cost estimates to the modeling of NICE, we can estimate that longer battery longevity might lead to savings of $2,629 to $6,977 per patient over 15 years; this would correspond to a total savings of $72.5 to $192.6 million. Of course, detailed cost analyses are beyond the scope of this paper and there are data suggesting that lowering initial device costs may eradicate the long-term cost benefits.^27^ These costs, however, do not account for the nonfinancial, clinical ramifications of additional complications resulting from more frequent generator changes and patient preferences for longer battery longevity.^20^ Finally, it is difficult to quantify the underlying systemic conflicts of interest where frequent CRT-D generator changes continue to drive fee-for-service or productivity-based reimbursements for physicians and health systems while, at the same time, increase costs and complication rates for payors (mainly Medicare) and patients.

### Limitations

We only examined outcomes of the 2.1-Ah CRT-D because this is the largest-capacity battery made available since 2008 for “real-world” outcomes analyses. Along these lines, **Table 3** shows details of commonly available CRT-D battery technology. We feel this limitation is counterbalanced by extensive prior analyses comparing device longevities that noted substantial differences in battery longevity conducted as part of outcomes-based research; here, larger-battery capacities were associated with improved device longevity.^5–8,21^ We must note that the larger 2.192-Ah battery device **(Table 3)** was not used in our facility during the study period and has only been available since 2015. We are unaware of any “real-world” outcomes data for this technology; rather, the longevity of the 2.192-Ah device has only been predicted to date. Compiling these “real-world” industry-independent, device longevity data is critical because “apart from the possible mismatch between real-life conditions and accelerated battery-life tests, data presented in the device manuals are difficult to interpret due to the lack of homogeneity in the assumptions for longevity projections and the paucity of information [available] about the impact of specific features on battery drain.”^3^ **Table 3** describes battery features and predicted longevities of currently available CRT-D systems. One can see the difference in total battery longevity (the nomenclature used in this study) versus “usable” battery capacity. Battery longevity is not only impacted by battery chemistry and capacity but also by advanced device features/algorithms. We did not include device pacing or lead parameters because prior studies have indicated that the total Ah capacity of the battery was the most consistent predictor of device longevity.^5–7^ Finally, each device has an intrinsic background housekeeping the current drain that must be back-calculated because it has not been provided by manufacturers.^4^ Lowering this housekeeping current drain can improve battery longevity. Again, our data provide no insight on “real-world” device longevity for the 2.192-Ah CRT-D with a lower background housekeeping current of 11.4 μA. Our analysis of the largest Ah-battery-capacity CRT-D that has been clinically available since 2008 was the only reasonable way to assess for reversal of the patient survival versus battery longevity mismatch.

Certainly, extended device longevity may not be as important in patients with shorter life expectancies or those under evaluation for heart transplantation. Likewise, device longevity may be even more important in younger patients with genetic channelopathies expected to have much longer survival estimates. Obviously, if our patient survival was substantially less than is typical, our battery longevity data may be skewed. Our data were from a large community hospital cohort with demographics (eg, age, creatinine, EF) similar (if not older) to those of other studies.^30–34^ During the mean follow-up period of 4.7 ± 2.7 years, there were 105 patient deaths; this translates to a 74.9% survival rate. There were no devices that were OOS due to ERI at less than the mean follow-up time of 4.7 years. This survival rate could be misleading because it does not take into account mortality that may have been experienced by patients censored after their initial device reached OOS during the study period. Furthermore, we did not examine hospitalization rates of patients in our retrospective review and recent data have suggested a 25% five-year survival of patients hospitalized with HFrEF.^35^ Our average age at implantation was 72.3 years and another study looking at survival did indicate an average age of death of 79 years.^35^ A large meta-analysis examining the survival of 1.5 million community CHF patients including mostly from studies in North America and Europe found a five-year survival rate of 59.7% with increased mortality in patients aged older than 75 years (versus those younger than 65 years).^15^ One of the most recent studies on mortality in stable outpatient CHF patients with reduced EF demonstrated a 66% survival rate with a mean follow-up of 39 months and an average age of 69 years.^36^ We feel that the overall survival of our patient population is consistent with that of a typical HFrEF cohort with an average life-expectancy comparable to the findings of prior studies.

Finally, the burden of arrhythmia, ventricular tachycardia/ventricular fibrillation therapies, and the New York Heart Association classification were not examined. The authors’ laboratory previously examined battery longevities across several manufacturers and used these prior data when developing the protocol for data acquisition in this study. In that study, we did not find a statistically significant difference in shock burden to explain the significant differences in battery longevity. Furthermore, although we did not examine the arrhythmia burden in our prior study,^5^ there was no statistically significant difference in the battery longevity despite variations in the percenage of biventricular pacing (91%–98%) across all manufacturers. One could infer that lower-percent biventricular pacing could result from a higher burden of arrhythmia. Of note, Alam et al.^7^ found that the 2.1-Ah battery was also associated with extended longevity even when controlling for “known parameters affecting battery drainage, including lead parameters and burden of pacing and tachyarrhythmia therapy.” Ultimately, we did not feel the lack of data regarding arrhythmia, ventricular tachycardia/ventricular fibrillation therapies, or New York Heart Association classification would limit our ability to study patient survival versus ICD battery longevity using the 2.1-Ah battery.

## Conclusions

To our knowledge, this is the first report demonstrating long-term patient outcomes (> 10 years in many cases) exclusively while using 2.1-Ah battery CRT-D technology. These data demonstrated the first reversal in ICD battery longevity versus patient survival; the 2.1-Ah ICD battery life exceeded patient survival in a typical HFrEF cohort. Patient deaths were a more frequent cause of CRT-D OOS (mean time to death: 2.3 ± 2.1 years) rather than ERI (mean time to ERI: 7.8 ± 1.5 years).

We found that male sex, ischemic cardiomyopathy, advanced age, elevated creatinine level, and lower EF were associated with CRT-D OOS and consistent with the findings of prior research on CHF patient survival. Our results support the hypothesis that the acceleration of device OOS during the sixth to ninth years (when it is expected that roughly 98% of 1.0-Ah and 1.4-Ah CRT-D systems reach ERI) may explain the historically high rate of complications for ICD generator changes as compared with at the initial implantation. During this accelerated time of CRT-D OOS, patient death (n = 105; 65.6%) resulted in numerically more instances of OOS than battery replacement (n = 24; 15%) and increased battery longevity avoided costs of complications and generator changes.

Our study sheds new light on battery longevity versus patient survival with HFrEF and these results can help explain the elevated risks of CRT-D generator changes demonstrated prior to battery longevity exceeding patient survival. More research is needed to examine the clinical and cost-effectiveness of avoiding generator changes during a vulnerable physiologic time in the lives of CRT-D patients.

## Figures and Tables

**Table 1:** Mode and Timing to OOS

	Number of Events (% of total OOS)	Time to OOS (mean ± SD years)
Advisory/recall	8 (5%)	5.3 ± 1.6
Elective replacement indicator	24 (15%)	7.8 ± 1.5
Heart transplant	1 (0.6%)	0.2
Infection/erosion	9 (5.6%)	1.5 ± 2.0
Patient death	105 (65.6%)	2.3 ± 2.1
Unspecified	13 (8.1%)	3.9 ± 2.6

ERI: elective replacement indicator; OOS: out of service; SD: standard deviation.

**Table 2:** Comparison Between Active Devices and Devices Reaching End of Service

	Device Active	Device OOS	HR	95% CI	p-value
Male sex	159/258 (61.6%)	113/160 (70.6%)	0.918	6.06–7.06	0.025
Ischemic cardiomyopathy	123/254 (48.4%)	100/160 (62.5%)	0.728	5.85–6.9	0.005
Age, years	70.9 ± 10.7	74.5 ± 10.6	1.03	72.8–76.1	0.005
Creatinine, mg/dL	1.2 ± 0.5	1.4 ± 0.6	1.551	1.3–1.5	0.031
EF	0.28 ± 0.11	0.25 ± 0.09	0.045	0.24–0.27	0.008

CI: confidence interval; EF: ejection fraction; HR: hazard ratio; OOS: out of service; SD: standard deviation.

Data are expressed as mean ± SD or n (%), as appropriate. Data for type of cardiomyopathy was not available for four patients. Baseline clinical characteristics were compared between patients with devices that reached OOS and those with active devices during follow-up using the chi-squared test for dichotomous variables and one-way multivariate analysis of variance and Tukey’s post-hoc test for continuous variables, as appropriate.

**Table 3:** Battery Features and Predicted Longevity of Current CRT-D Systems

Device	Battery Chemistry	Usable Battery Energy	Total Battery Capacity/to RRT	Average Housekeeping Current	Predicted Longevity
Sorin Platinium 4LV SonR	Hybrid Li-CFx/LiSVO	1.33 Ah	2.192 Ah/1.53 Ah	11.4 μA	13.1 years
Boston Scientific Resonate X4	Li-MnO2	1.3 Ah	2.1 Ah/1.75 Ah	18.3 μA	11.6 years
St. Jude/Abbott Quadra Assura MP	Hybrid Li-CFx/Li-SVO	1.13 Ah	1.944 Ah/1.377 Ah	15.4 μA	8.4 years
Biotronik Intica 7 HF-T (QP)	Hybrid Li-CFx/Li-SVO	0.907 Ah	1.73 Ah/1.52 Ah	18.4 μA	7.8 years
Medtronic Viva Quad XT	Li-CFx/Li-SVO	0.664 Ah	1.0 Ah*	13.0 μA	7 years

CRT: cardiac resynchronization; ICD: implantable cardioverter defibrillator; RRT: recommended replacement time; Li-CFx: lithium–carbon mono-fluoride; Li-SVO: lithium–silver vanadium oxide; Li-MnO_2_: lithium manganese dioxide.

Data are adapted from Munawar et al.^4^ and Lau^29^ with predicted longevity standardized to the setting in the Viva Quad XT (Medtronic, Minneapolis, MN, USA) manual.

*Total battery capacity for Viva Quad XT not available; 1.0-Ah capacity to RRT.
